# *N*-[2-(Phenyl­sulfon­yl)eth­yl]benzyl­amine^1^

**DOI:** 10.1107/S160053681203886X

**Published:** 2012-09-15

**Authors:** David A. Aubry, Frank R. Fronczek, Steven F. Watkins

**Affiliations:** a Department of Chemistry, Louisiana State University, Baton Rouge, LA 70803-1804, USA

## Abstract

The title compound, C_15_H_17_NO_2_S, exhibits intra­molecular hydrogen bonding between the amine H atom and a sulfonyl O atom. The conformation of the mol­ecule is described by the four PhCH_2_—NH—CH_2_—CH_2_—SO_2_Ph torsion angles of 79.6 (2), −166.21 (14), −70.29 (17) and −58.93 (13)°.

## Related literature

For the synthesis, see: Bandini *et al.* (2008 ▶). For reactions of benzyl­amine and vinyl­sulfonyl­benzene, see: Makosza *et al.* (2008 ▶); Ni *et al.* (2003 ▶). For the determination of absolute structure from Bijvoet pairs, see: Hooft *et al.* (2008 ▶). For inter­molecular inter­actions, see: Steiner (1996 ▶).
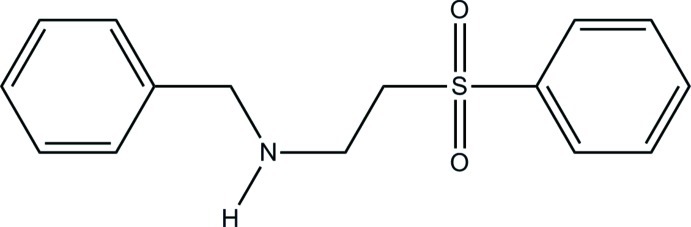


## Experimental

### 

#### Crystal data

C_15_H_17_NO_2_S
*M* *_r_* = 275.36
Monoclinic, 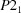

*a* = 5.7428 (11) Å
*b* = 10.170 (2) Å
*c* = 12.486 (2) Å
β = 102.09 (3)°
*V* = 713.1 (2) Å^3^
*Z* = 2
Mo *K*α radiation
μ = 0.22 mm^−1^
*T* = 298 K
0.50 × 0.45 × 0.32 mm

#### Data collection

Enraf–Nonius CAD-4 diffractometer
Absorption correction: ψ scan (North *et al.*, 1968 ▶) *T*_min_ = 0.896, *T*_max_ = 0.932
5320 measured reflections
3264 independent reflections
2990 reflections with *I* > 2σ(*I*)
*R*_int_ = 0.021
3 standard reflections every 60 min intensity decay: 4.0%

#### Refinement

*R*[*F* ^2^ > 2σ(*F* ^2^)] = 0.029
*wR*(*F* ^2^) = 0.086
*S* = 0.99
3264 reflections
177 parameters
1 restraint
H atoms treated by a mixture of independent and constrained refinement
Δρ_max_ = 0.15 e Å^−3^
Δρ_min_ = −0.22 e Å^−3^
Absolute structure: Flack (1983 ▶), 2081 Bijvoet pairs
Flack parameter: 0.07 (6)

### 

Data collection: *CAD-4 EXPRESS* (Enraf–Nonius, 1994 ▶); cell refinement: *CAD-4 EXPRESS*; data reduction: *XCAD4* (Farrugia, 1999 ▶); program(s) used to solve structure: *SIR2002* (Burla *et al.*, 2003 ▶); program(s) used to refine structure: *SHELXL97* (Sheldrick, 2008 ▶); molecular graphics: *ORTEP-3 for Windows* (Farrugia, 1997 ▶); software used to prepare material for publication: *WinGX* (Farrugia, 1999 ▶).

## Supplementary Material

Crystal structure: contains datablock(s) global, I. DOI: 10.1107/S160053681203886X/bg2477sup1.cif

Structure factors: contains datablock(s) I. DOI: 10.1107/S160053681203886X/bg2477Isup2.hkl

Supplementary material file. DOI: 10.1107/S160053681203886X/bg2477Isup3.cml

Additional supplementary materials:  crystallographic information; 3D view; checkCIF report

## Figures and Tables

**Table 1:** Hydrogen-bond geometry (Å, °)

*D*—H⋯*A*	*D*—H	H⋯*A*	*D*⋯*A*	*D*—H⋯*A*
N1—H1⋯O1	0.87 (2)	2.52 (2)	3.071 (2)	121.9 (17)

**Fig. 1.**
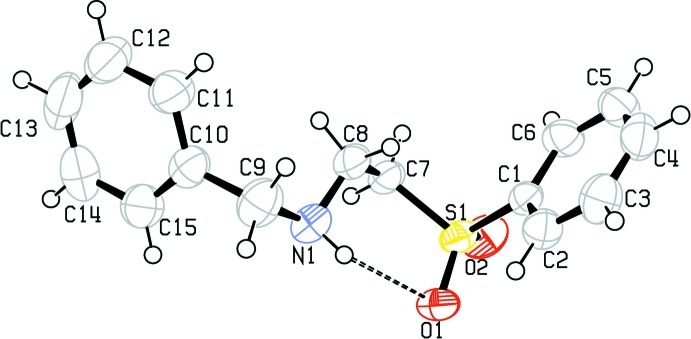
View of (I) (50% probability displacement ellipsoids)

## supplementary crystallographic information

### Comment

The most striking feature of title compound (I), a secondary amine, is the intramolecular hydrogen bond between a sulfonyl oxygen and the amine hydrogen. The complete geometry of this system is: N—H = 0.87 (2) Å, H···O = 2.52 (2) Å, N—H···O = 122 (2)°, S═O···H = 97 (2)°. The two six-rings are flat: C1—C6, δ_r.m.s._ = 0.0049 Å, C10—C15, δ_r.m.s._ = 0.0024 Å. The absolute structure was determined by analysis of 2081 Bijvoet pairs.

In addition to the intramolecular hydrogen bond, weaker intermolecular C—H···O and C—H···π interactions (Steiner, 1996) are present. The geometry of the C—H···O interaction is C7–H7A···O1 (at *x* + 1, *y*, *z*), C7···O1 3.3896 (18) Å, H7A···O1 2.44 Å, angle about H 166°, forming chains in the [100] direction. The C—H···π interaction involves the phenyl C4—H as donor and the other phenyl ring C10—C15 (at 1 - *x*, *y* - 1/2, 1 - *z*) as acceptor. The C4···*Cg* distance is 3.731 (2) Å, and the contact is fairly linear, with angle about H 174°. This interaction forms chains in the [010] direction, propagated by the screw axis.

### Experimental

This compound was synthesized from benzylamine and vinylsulfonylbenzene by Dr. J. Gabriel Garcia. It was recrystallized by very slow cooling of an ethanol solution.

### Refinement

The coordinates and isotropic displacement parameter of the amine hydrogen atom were refined without constraint. All other H atoms were placed in calculated positions with C(*sp*^2^)—H = 0.930 Å, C(*sp*^3^)—H = 0.970 Å, *U*_iso_(H) = 1.2*U*_eq_(C), and thereafter allowed to ride the attached C atom. The absolute structure is supported by analysis of 2081 Bijvoet pairs, with Flack (1983) parameter *x* = 0.07 (6) and Hooft (Hooft *et al.*, 2008) parameter *y* = 0.12 (2).

### Crystal data

**Table:**

C_15_H_17_NO_2_S	*F*(000) = 292
*M**_r_* = 275.36	*D*_x_ = 1.282 Mg m^−^^3^
Monoclinic, *P*2_1_	Mo *K*α radiation, λ = 0.71073 Å
Hall symbol: P 2yb	Cell parameters from 25 reflections
*a* = 5.7428 (11) Å	θ = 2.6–27.5°
*b* = 10.170 (2) Å	µ = 0.22 mm^−^^1^
*c* = 12.486 (2) Å	*T* = 298 K
β = 102.09 (3)°	Prism, colorless
*V* = 713.1 (2) Å^3^	0.50 × 0.45 × 0.32 mm
*Z* = 2	

### Data collection

**Table:**

Enraf–Nonius CAD-4 diffractometer	2990 reflections with *I* > 2σ(*I*)
Radiation source: fine-focus sealed tube	*R*_int_ = 0.021
Graphite monochromator	θ_max_ = 27.5°, θ_min_ = 2.6°
θ/2θ scans	*h* = −7→7
Absorption correction: ψ scan (North *et al.*, 1968)	*k* = −13→13
*T*_min_ = 0.896, *T*_max_ = 0.932	*l* = −16→16
5320 measured reflections	3 standard reflections every 60 min
3264 independent reflections	intensity decay: 4.0%

### Refinement

**Table:**

Refinement on *F*^2^	Secondary atom site location: difference Fourier map
Least-squares matrix: full	Hydrogen site location: inferred from neighbouring sites
*R*[*F*^2^ > 2σ(*F*^2^)] = 0.029	H atoms treated by a mixture of independent and constrained refinement
*wR*(*F*^2^) = 0.086	*w* = 1/[σ^2^(*F*_o_^2^) + (0.0562*P*)^2^ + 0.0525*P*] where *P* = (*F*_o_^2^ + 2*F*_c_^2^)/3
*S* = 0.99	(Δ/σ)_max_ < 0.001
3264 reflections	Δρ_max_ = 0.15 e Å^−^^3^
177 parameters	Δρ_min_ = −0.22 e Å^−^^3^
1 restraint	Absolute structure: Flack (1983), 2081 Bijvoet pairs
0 constraints	Flack parameter: 0.07 (6)
Primary atom site location: structure-invariant direct methods	

### Special details

**Table:**

Geometry. All e.s.d.'s (except the e.s.d. in the dihedral angle between two l.s. planes) are estimated using the full covariance matrix. The cell e.s.d.'s are taken into account individually in the estimation of e.s.d.'s in distances, angles and torsion angles; correlations between e.s.d.'s in cell parameters are only used when they are defined by crystal symmetry. An approximate (isotropic) treatment of cell e.s.d.'s is used for estimating e.s.d.'s involving l.s. planes.

### Fractional atomic coordinates and isotropic or equivalent isotropic displacement parameters (Å^2^)

**Table:**

	*x*	*y*	*z*	*U*_iso_*/*U*_eq_	
C1	0.1957 (3)	0.01813 (15)	0.34724 (12)	0.0424 (3)	
C2	0.0482 (3)	−0.08893 (17)	0.34717 (13)	0.0533 (4)	
H2	−0.0657	−0.0884	0.3903	0.064*	
C3	0.0698 (4)	−0.1963 (2)	0.28342 (15)	0.0657 (5)	
H3	−0.0305	−0.2682	0.2826	0.079*	
C4	0.2404 (4)	−0.1973 (2)	0.22054 (14)	0.0639 (5)	
H4	0.254	−0.2699	0.177	0.077*	
C5	0.3912 (4)	−0.0917 (2)	0.22157 (15)	0.0656 (5)	
H5	0.5078	−0.094	0.1799	0.079*	
C6	0.3693 (3)	0.01759 (19)	0.28437 (14)	0.0561 (4)	
H6	0.469	0.0897	0.2847	0.067*	
C7	0.4077 (2)	0.1536 (2)	0.53880 (12)	0.0522 (3)	
H7A	0.5518	0.1629	0.5105	0.063*	
H7B	0.3977	0.2293	0.5848	0.063*	
C8	0.4311 (3)	0.03164 (18)	0.60956 (14)	0.0560 (4)	
H8A	0.4215	−0.0452	0.5628	0.067*	
H8B	0.587	0.0316	0.658	0.067*	
C9	0.3093 (4)	−0.0788 (2)	0.76111 (17)	0.0691 (5)	
H9A	0.374	−0.1558	0.732	0.083*	
H9B	0.1651	−0.1049	0.7843	0.083*	
C10	0.4876 (3)	−0.02798 (17)	0.85863 (14)	0.0554 (4)	
C11	0.7095 (4)	−0.0864 (2)	0.88806 (17)	0.0698 (5)	
H11	0.7488	−0.156	0.8469	0.084*	
C12	0.8722 (4)	−0.0421 (3)	0.97776 (19)	0.0892 (8)	
H12	1.021	−0.0819	0.9964	0.107*	
C13	0.8185 (5)	0.0584 (3)	1.03905 (17)	0.0914 (8)	
H13	0.9293	0.0868	1.1	0.11*	
C14	0.6002 (5)	0.1187 (3)	1.01140 (17)	0.0836 (7)	
H14	0.5637	0.1887	1.0529	0.1*	
C15	0.4339 (4)	0.0746 (2)	0.92103 (16)	0.0670 (5)	
H15	0.2854	0.1147	0.9027	0.08*	
N1	0.2511 (3)	0.02105 (18)	0.67505 (12)	0.0610 (4)	
H1	0.122 (4)	−0.001 (2)	0.6295 (17)	0.059 (5)*	
O1	−0.05605 (19)	0.13963 (16)	0.46634 (10)	0.0646 (4)	
O2	0.1828 (3)	0.27332 (14)	0.36372 (13)	0.0725 (4)	
S1	0.16006 (6)	0.15714 (4)	0.42668 (3)	0.04876 (11)	

### Atomic displacement parameters (Å^2^)

**Table:**

	*U*^11^	*U*^22^	*U*^33^	*U*^12^	*U*^13^	*U*^23^
C1	0.0372 (7)	0.0472 (8)	0.0405 (6)	0.0003 (6)	0.0032 (5)	0.0037 (6)
C2	0.0498 (8)	0.0577 (9)	0.0529 (8)	−0.0103 (7)	0.0121 (7)	0.0007 (7)
C3	0.0720 (11)	0.0584 (10)	0.0625 (10)	−0.0135 (9)	0.0046 (9)	−0.0038 (8)
C4	0.0772 (12)	0.0615 (10)	0.0479 (8)	0.0123 (9)	0.0017 (8)	−0.0040 (8)
C5	0.0635 (10)	0.0825 (13)	0.0540 (9)	0.0153 (10)	0.0195 (8)	0.0043 (9)
C6	0.0481 (8)	0.0630 (10)	0.0598 (9)	−0.0047 (7)	0.0170 (7)	0.0065 (8)
C7	0.0383 (6)	0.0595 (8)	0.0546 (7)	0.0006 (9)	0.0004 (5)	−0.0105 (9)
C8	0.0480 (8)	0.0631 (10)	0.0521 (8)	0.0157 (7)	−0.0006 (7)	−0.0062 (7)
C9	0.0672 (11)	0.0664 (11)	0.0689 (11)	−0.0122 (9)	0.0035 (9)	0.0035 (9)
C10	0.0537 (9)	0.0578 (9)	0.0535 (8)	−0.0053 (7)	0.0082 (7)	0.0125 (7)
C11	0.0607 (10)	0.0811 (13)	0.0670 (11)	0.0066 (10)	0.0123 (9)	0.0108 (10)
C12	0.0620 (12)	0.129 (2)	0.0703 (13)	0.0054 (13)	0.0007 (10)	0.0235 (15)
C13	0.0837 (16)	0.133 (2)	0.0498 (10)	−0.0307 (16)	−0.0048 (10)	0.0129 (13)
C14	0.1094 (19)	0.0896 (16)	0.0552 (10)	−0.0127 (13)	0.0251 (11)	−0.0065 (10)
C15	0.0675 (12)	0.0706 (12)	0.0636 (10)	0.0038 (9)	0.0152 (9)	0.0075 (9)
N1	0.0474 (8)	0.0746 (10)	0.0560 (8)	0.0048 (7)	−0.0005 (6)	−0.0003 (7)
O1	0.0386 (5)	0.0823 (10)	0.0720 (7)	0.0089 (6)	0.0094 (5)	−0.0116 (7)
O2	0.0716 (9)	0.0513 (7)	0.0871 (9)	0.0056 (6)	−0.0010 (7)	0.0134 (7)
S1	0.03744 (16)	0.04880 (18)	0.05641 (19)	0.00474 (16)	0.00158 (12)	0.00009 (18)

### Geometric parameters (Å, º)

**Table:**

C1—C2	1.379 (2)	C9—N1	1.465 (3)
C1—C6	1.392 (2)	C9—C10	1.508 (3)
C1—S1	1.7634 (16)	C9—H9A	0.97
C2—C3	1.372 (3)	C9—H9B	0.97
C2—H2	0.93	C10—C15	1.375 (3)
C3—C4	1.378 (3)	C10—C11	1.384 (3)
C3—H3	0.93	C11—C12	1.376 (3)
C4—C5	1.378 (3)	C11—H11	0.93
C4—H4	0.93	C12—C13	1.351 (4)
C5—C6	1.381 (3)	C12—H12	0.93
C5—H5	0.93	C13—C14	1.373 (4)
C6—H6	0.93	C13—H13	0.93
C7—C8	1.512 (3)	C14—C15	1.391 (3)
C7—S1	1.7748 (15)	C14—H14	0.93
C7—H7A	0.97	C15—H15	0.93
C7—H7B	0.97	N1—H1	0.87 (2)
C8—N1	1.450 (2)	O1—S1	1.4404 (12)
C8—H8A	0.97	O2—S1	1.4402 (15)
C8—H8B	0.97		
			
C2—C1—C6	120.57 (16)	C10—C9—H9A	109.3
C2—C1—S1	119.32 (12)	N1—C9—H9B	109.3
C6—C1—S1	120.10 (13)	C10—C9—H9B	109.3
C3—C2—C1	119.89 (16)	H9A—C9—H9B	108
C3—C2—H2	120.1	C15—C10—C11	118.58 (19)
C1—C2—H2	120.1	C15—C10—C9	121.49 (18)
C2—C3—C4	119.87 (18)	C11—C10—C9	119.93 (19)
C2—C3—H3	120.1	C12—C11—C10	120.4 (2)
C4—C3—H3	120.1	C12—C11—H11	119.8
C5—C4—C3	120.62 (18)	C10—C11—H11	119.8
C5—C4—H4	119.7	C13—C12—C11	120.8 (2)
C3—C4—H4	119.7	C13—C12—H12	119.6
C4—C5—C6	120.05 (17)	C11—C12—H12	119.6
C4—C5—H5	120	C12—C13—C14	120.0 (2)
C6—C5—H5	120	C12—C13—H13	120
C5—C6—C1	118.99 (16)	C14—C13—H13	120
C5—C6—H6	120.5	C13—C14—C15	119.7 (2)
C1—C6—H6	120.5	C13—C14—H14	120.2
C8—C7—S1	115.86 (13)	C15—C14—H14	120.2
C8—C7—H7A	108.3	C10—C15—C14	120.5 (2)
S1—C7—H7A	108.3	C10—C15—H15	119.8
C8—C7—H7B	108.3	C14—C15—H15	119.8
S1—C7—H7B	108.3	C8—N1—C9	112.65 (15)
H7A—C7—H7B	107.4	C8—N1—H1	105.3 (13)
N1—C8—C7	113.80 (13)	C9—N1—H1	109.5 (14)
N1—C8—H8A	108.8	O2—S1—O1	118.19 (9)
C7—C8—H8A	108.8	O2—S1—C1	108.44 (8)
N1—C8—H8B	108.8	O1—S1—C1	107.70 (8)
C7—C8—H8B	108.8	O2—S1—C7	107.33 (10)
H8A—C8—H8B	107.7	O1—S1—C7	109.39 (8)
N1—C9—C10	111.51 (16)	C1—S1—C7	105.02 (8)
N1—C9—H9A	109.3		
			
C6—C1—C2—C3	−1.0 (3)	C12—C13—C14—C15	0.9 (4)
S1—C1—C2—C3	178.24 (14)	C11—C10—C15—C14	0.3 (3)
C1—C2—C3—C4	0.7 (3)	C9—C10—C15—C14	179.22 (18)
C2—C3—C4—C5	0.5 (3)	C13—C14—C15—C10	−0.7 (3)
C3—C4—C5—C6	−1.2 (3)	C7—C8—N1—C9	−166.21 (14)
C4—C5—C6—C1	0.9 (3)	C10—C9—N1—C8	79.6 (2)
C2—C1—C6—C5	0.2 (2)	C2—C1—S1—O2	−138.39 (14)
S1—C1—C6—C5	−179.02 (13)	C6—C1—S1—O2	40.86 (14)
S1—C7—C8—N1	−70.29 (17)	C2—C1—S1—O1	−9.40 (15)
N1—C9—C10—C15	64.2 (2)	C6—C1—S1—O1	169.85 (13)
N1—C9—C10—C11	−116.9 (2)	C2—C1—S1—C7	107.12 (14)
C15—C10—C11—C12	−0.1 (3)	C6—C1—S1—C7	−73.64 (14)
C9—C10—C11—C12	−179.06 (19)	C8—C7—S1—O2	−174.19 (12)
C10—C11—C12—C13	0.4 (4)	C8—C7—S1—O1	56.42 (15)
C11—C12—C13—C14	−0.7 (4)	C8—C7—S1—C1	−58.93 (13)

### Hydrogen-bond geometry (Å, º)

**Table:**

*D*—H···*A*	*D*—H	H···*A*	*D*···*A*	*D*—H···*A*
N1—H1···O1	0.87 (2)	2.52 (2)	3.071 (2)	121.9 (17)
